# ^31^P NMR 2D Mapping of Creatine Kinase Forward Flux Rate in Hearts with Postinfarction Left Ventricular Remodeling in Response to Cell Therapy

**DOI:** 10.1371/journal.pone.0162149

**Published:** 2016-09-08

**Authors:** Ling Gao, Weina Cui, Pengyuan Zhang, Albert Jang, Wuqiang Zhu, Jianyi Zhang

**Affiliations:** 1 Department of Biomedical Engineering, School of Medicine and School of Engineering, University of Alabama at Birmingham, Birmingham, Alabama, 35233, United States of America; 2 Department of Medicine, Cardiology Division, University of Minnesota—Twin Cities, Minneapolis, Minnesota, 55455, United States of America; Georgia Regents University, UNITED STATES

## Abstract

Utilizing a fast ^31^P magnetic resonance spectroscopy (MRS) 2-dimensional chemical shift imaging (2D-CSI) method, this study examined the heterogeneity of creatine kinase (CK) forward flux rate of hearts with postinfarction left ventricular (LV) remodeling. Immunosuppressed Yorkshire pigs were assigned to 4 groups: 1) A sham-operated normal group (SHAM, n = 6); 2) A 60 minutes distal left anterior descending coronary artery ligation and reperfusion (MI, n = 6); 3) Open patch group; ligation injury plus open fibrin patch over the site of injury (Patch, n = 6); and 4) Cell group, hiPSCs-cardiomyocytes, -endothelial cells, and -smooth muscle cells (2 million, each) were injected into the injured myocardium pass through a fibrin patch (Cell+Patch, n = 5). At 4 weeks, the creatine phosphate (PCr)/ATP ratio, CK forward flux rate (Flux _PCr→ATP_), and k constant of CK forward flux rate (*k*_PCr→ATP_) were severely decreased at border zone myocardium (BZ) adjacent to MI. Cell treatment results in significantly increase of PCr/ATP ratio and improve the value of *k*_PCr→ATP_ and Flux _PCr→ATP_ in BZ myocardium. Moreover, the BZ myocardial CK total activity and protein expression of CK mitochondria isozyme and CK myocardial isozyme were significantly reduced, but recovered in response to cell treatment. Thus, cell therapy results in improvement of BZ bioenergetic abnormality in hearts with postinfarction LV remodeling, which is accompanied by significantly improvements in BZ CK activity and CK isozyme expression. The fast 2D ^31^P MR CSI mapping can reliably measure the heterogeneity of bioenergetics in hearts with post infarction LV remodeling.

## Introduction

Myocardial ATP demand per minute exceeds the amount of ATP present in myocardium of the heart by up to four orders of magnitude [1]; thus, the ATP machinery must be able to continually resynthesize amount of ATP just as much as it can be utilized at contractile apparatus to maintain a constant myocardial ATP concentration at different cardiac work states. Fatty acid oxidation in the mitochondria produces the majority of ATP to meet energy demand under normal and basal cardiac workloads. The additional mechanisms of ATP production are activated under high-cardiac workload conditions, including the phosphotransferase activities [2, 3]. Creatine kinase (CK) is a major source of phosphotransferase pathway that buffers ATP demand and maintain a constant ATP concentration at onset of the cardiac workload increase. CK rapidly and reversibly catalyzes the conversion of ADP and phosphocreatine (PCr) to ATP and creatine [2, 3] (i.e., the PCr→ATP reaction). Both clinical and experimental studies indicate that the CK expression level and activity are altered in LV hypertrophied (LVH) heart, which is most severe in LVH and failing hearts [4–7]. ^31^P magnetic resonance spectroscopy (^31^P-MRS) magnetization saturation transfer (MST) experiments have been used to measure the myocardial bioenergetics and myocardial the rate of CK forward flux (*Flux*_*PCr*→ATP_) in the in vivo hearts [1, 7–9]. Using a porcine model of postinfarction LV remodeling, Ye Y et al also showed that the PCr/ATP ratio and *k*_PCr→ATP_ are significantly lower in postinfarction LV remodeling heart, which is linearly related to the severity of LV hypertrophy, and LV chamber function that measured by cardiac MRI [7]. The conventional ^31^P-MRS MST method requires lengthy data acquisition time, which prohibit the spatial localization measurements of CK flux on a heterogeneous heart with postinfarction LV remodeling [10]. However, Xiong et al recently demonstrated mathematically and experimentally that by a novel T1^nom^ MRS method, the data acquisition time can be decreased to 18% of that of conventional methods [6, 9]. Furthermore, by placing the NMR coil on the different areas of hearts with postinfarction LV remodeling, these investigators demonstrated a remarkable heterogeneity of myocardial PCr/ATP ratio in a heart with compensated postinfarction LV remodeling with the surprisingly severe reduction in the BZ [11]. However, comparing the heterogeneity of CK forward flux rate of an in vivo heart with postinfarction LV remodeling by one set of NMR data acquisition have never been measured before. For the experiments described in this report, we combined ^31^P MRS-MST with 2-dimensional chemical-shift imaging (2D-CSI), which enabled us to obtain bioenergetic measurements over a range of sites extending from the infarct scarred zone (IZ), to periscar boarder zone (BZ), and non-ischemic remote zone (RZ), during one single set data acquisition period and without moving the NMR probe. We used this technique to determine whether a cell-free fibrin patch, either alone or when combined with injected human induced pluripotent stem cell (hiPSC)-ECs, -SMCs, and -CMs, improved bioenergetic parameters and CK activity in a swine ischemia reperfusion (IR) injury model.

## Materials and Methods

### Generation and characterization of hiPSC-derived tri lineage cardiac cells

The hiPSCs were generated by transfecting male human neonatal dermal fibroblasts with lentiviruses coding for OCT4, SOX2, KLF4, and C-MYC and then engineered to constitutively express green fluorescent protein (GFP) [12]. Cells were cultured in Matrigel-coated plates with mTeSR-1 culture medium (Stemcell Tech, USA) and passaged every 4–5 days. The protocols used to differentiate hiPSCs into hiPSC-ECs, -SMCs, and CMs have been described previously [13–16]. Briefly, undifferentiated hiPSCs were treated with the glycogen synthase kinase 3β inhibitor CHIR99021 and ascorbic acid for 5 days to induce mesoderm differentiation; then, CD34^+^ cells were collected via magnetic nano-particle selection and cultured on fibronectin-coated plates for differentiation into ECs, or on collagen IV-coated dishes for differentiation into SMCs. EC differentiation was induced by culturing the cells with EGM 2-MV medium (Lonza, USA) containing vascular endothelial growth factor and SB431542, and the hiPSC-ECs were purified to >95% by using a fluorescence-activated cell sorter (FACS) to collect cells that expressed both CD31 and CD144. SMC differentiation was induced by culturing the cells with SmGM-2 medium (Lonza, USA) containing platelet-derived growth factor BB and transforming growth factor β. For differentiation into hiPSC-CMs [14], the hiPSCs were expanded on a Matrigel-coated dish for 4 days; then, differentiation was induced by culturing the cells with CHIR99021 in RPMI basal medium plus B27 without insulin (B27^–^). Twenty-four hours later, the cells were recovered and cultured with RPMI basal medium plus B27^–^ for 2 days; then, the cells were cultured in RPMI basal medium with B27^–^ and the Wnt-signaling inhibitor IWP-2. Beating cells usually appeared about 8 days after differentiation was initiated, and the hiPSC-CMs were purified and enriched to >95% via metabolic selection [17]. hiPSC-CMs were characterized via the expression of cardiac troponin I (cTnI), cardiac troponin T (cTnT), α-sarcomeric actin (αSA), connexin 43 (Con43), and ventricular myosin light chain 2 (MLC-2v). hiPSC-ECs were differentiated and characterized as previously described in details via the expression of CD31, CD144, and von Willebrand factor-8 [16]. hiPSC-SMCs were differentiated and characterized via the expression of α-smooth muscle actin (αSMA), smooth muscle 22α (SM22), and calponin as previously described in details [18].

### Porcine IR injury model and cell transplantation

All procedures and protocols involving animals were approved by the Institutional Animal Care and Use Committee of the University of Alabama at Birmingham and performed in accordance with the National Research Council’s Guide for the Care and Use of Laboratory Animals. Experiments were performed with female Yorkshire swine (14 kg, 45 days of age, Manthei hog farm, Elk River, MN), and the animals were randomly divided into 4 experimental groups. Animals in the Cell + Patch group (n = 5) were treated with 2 million hiPSC-CMs, 2 million hiPSC-ECs, and 2 million hiPSC-SMCs (6 million cells total), which were injected directly into the injured myocardium through a fibrin patch that had been created over the site of injury. Animals in the Patch group (n = 6) were treated with the patch alone, and both treatments were withheld from animals in the MI group (n = 6). Animals in the Sham group (n = 6) underwent all surgical procedures for the induction of IR injury except for the coronary ligation step. Myocardial IR injury was induced as described previously [11, 19–21]. Briefly, animals were anesthetized with inhaled 2% isoflurane, intubated, and ventilated with a respirator and supplemental oxygen. Body temperature, electrocardiograms, blood pressure, and arterial oxygen saturation were monitored throughout the surgical procedure. A left thoracotomy was performed, and the root of the first and second diagonal coronary arteries from the left anterior descending coronary artery (LAD) was occluded for 60 minutes before reperfusion and treatment administration; then, the chest was closed in layers, and the animal was allowed to recover. Standard post-operative care, including analgesia, was administered until the animals ate normally and became active. General surgical anesthesia is monitored by the assistant surgeon and the individual circulating in the operation room. Surgical anesthesia is judged by the loss of muscle tone in the jaw and limbs, lack of spontaneous movement in response to noxious stimuli, loss of corneal reflex, and absence spontaneous respiration. the ECG, HR and T will be monitored by the life support system at the same time. Throughout the surgical period, animals will be kept on a warm, padded table with drapes over them. After the surgery is completed, animal are transferred to the post-surgical care unit. Care in this facility is supervised by veterinary staff. In addition, animals are seen and examined on a daily basis by a member of our research staff. Because the transplanted cells were derived from human cells, animals in all treatment groups received cyclosporine (15 mg/kg per day with food) for immune-suppression.

The fibrin patch was created by co-injecting 1 mL fibrinogen solution (25 mg/mL) with 1 mL thrombin solution (80 NIH units/mL, supplemented with 2 μL 400 mM CaCl_2_ and 200 mM Ɛ-aminocaproic acid) into a plastic ring (2.3-cm diameter) that had been placed on the epicardium of the infarcted region to serve as a mold for the patch [11]; the mixture usually solidified within 30 seconds

At the end of experiment, under anaesthesia condition swines were sacrificed by injecting potassium chloride (2 mEq/kg) through vein, and the thoracotomy and cardiectomy were performed to harvest the hearts

### Contractile function and infarct size measurement by MRI

Magnetic Resonance Imaging (MRI) was performed to assess cardiac functional outcome and infarct size on a 1.5 Tesla clinical scanner (Siemens Sontata, Siemens Medical Systems, Germany) with a phased-array 4-channel surface coil and ECG gating [9, 12, 22]. Animals were anesthetized with 2% inhaled isoflurane and positioned in a supine position within the scanner, and measurements of cardiac function were determined from short-axis cine images by using the QMASS program (Medis Medical Imaging Systems, The Netherlands). Cine imaging was performed with the following MR parameters: TR = 3.1 ms, TE = 1.6 ms, flip angle = 79°, matrix size = 256×120, field of view = 340×265 mm^2^, slice thickness = 6 mm (with a 4-mm gap between slices); 25 phases were acquired across the cardiac cycle. Infarct size was measured via delayed enhancement MRI (0.2 mmol/kg gadopentetate dimeglumine, iv, bolus) and quantified with ImageJ software (National Institutes of Health). Delayed enhancement MRI was acquired with the following parameters: TR = 16 ms, TE = 4 ms, TI = 300 ms, flip angle = 30°, matrix size = 256×148, field of view = 320×185 mm^2^, slice thickness = 6 mm (with a 0-mm gap between slices).

### Hemodynamic and wall stress assessments

Animals were anesthetized with 2% isoflurane and ventilated with air and supplemental oxygen (1:1 ratio) on a respirator. Two polyvinyl chloride catheters (3-mm outer diameter) were inserted into the vessels of the animal, one was placed in jugular vein, and the other was inserted in ascending aorta (through the left external carotid artery) and advanced to left ventricle for hemodynamic monitoring as previously described [11, 12]. Ventilation rate, volume, and inspired oxygen content were adjusted to maintain physiological values for arterial pO_2_, pCO_2_, and pH. Aortic and LV pressures were continuously monitored with a PowerLab system (AD Instrument, NSW, Australia) throughout the study. Wall stress was calculated from the cardiac MRI and hemodynamic data according to the Laplace model [23, 24]:

Systolic Wall stress=LV systolic pressure×LV chamber radius/(2×LV thickness)

### In vivo cardiac ^31^P MRS assessments

In vivo measurements of myocardial bioenergetics were performed in a 9.4T/65-cm bore magnet via the T_1_^Nom 31^P MRS and 2-dimensional chemical shift imaging (^31^P-2D-CSI) as previously described in details [6, 9, 12, 19]. The infarct and peri-infarct region was identified as the open chest preparation expose the heart; then, a double-tuned (^1^H/^31^P) surface coil (28 mm in diameter) was sutured directly to the epicardium covering both IZ and BZ region. The proton signal from water was detected with the surface coil and used to adjust the position of the animal in the magnet so that the coil was at the magnetic isocenter; then, the magnetic field to be homogenized for acquisition of the anatomic information needed to plan the ^31^P-2D-CSI experiments. ^31^P MR spectra were acquired with an adiabatic half-passage pulse to minimize any variation in the flip-angle that may have been caused by B1 inhomogeneity from the surface coil. The ^31^P-2D-CSI assessments consisted of 10×8 phase encoding steps, which covered a field of view of 5×4 cm^2^ and provided a spatial resolution of 0.5×0.5 cm^2^. Acquisition was gated to both the cardiac and respiratory cycles, and the average repetition time (TR) was 2.7 sec [11]. Each phase encoding step comprised 12 repetitive scans, and the total time required for data acquisition was 43 min. The raw data were processed via Fourier series windowing reconstruction with a home-built Matlab program, and the PCr and ATPγ peaks were integrated for each voxel. Two global spectra (with TR = 2.7 s and TR = 12 s) were also collected and used to correct calculations of the PCr/ATPγ ratio for partial saturation. The rates of phosphocreatine-mediated ATP production (*k*_PCr→ATP_) were calculated from the following equations. The accuracy and robustness of the optimized MST method have been demonstrated by the computer simulation and rigorous experimental testing has been reported recently in details [6, 11, 19].

$$k_{\text{PCr}\to \text{ATP}}=\left(\frac{M_{0,PCr}-M_{SS,PCr}}{M_{SS,PCr}}\right)/T_{1,PCr}^{int}$$

Here M represents the fully relaxed magnetizations of PCr and ATPγ with (M_SS_) and without (M_0_) saturation of ATPγ, and T_1_^int^ is the intrinsic longitudinal relaxation time constant. The CK forward flux rate (Flux _PCr→ATP_) was calculated as the products of *k*_PCr→ATP_ and myocardial PCr level [11, 25]. PCr values obtained from the PCr/ATP of the spectra and chemically determined ATP levels by using a fluorometric ATP Assay Kit (ThermoFisher Scientific, USA) [19].

### CK isozyme expression levels and ex-vivo activity

Full-thickness myocardial samples were taken from the BZ and frozen in liquid nitrogen. CK protein levels were quantified via Western blotting as described previously [7, 26, 27]. Briefly, protein extracts were run on 10% SDS-PAGE gels in a Protean Electrophoresis apparatus (BioRad, USA), followed by 1 hour of transferred in transfer buffer (25 mM Tris, 192 mM glycine, 20% methanol), and then labeled with isoform-specific antibodies against myocardial, brain, and mitochondrial CK (CK-M, CK-B, and CK-MT, respectively) (CK-M and CK-B antibodies: mouse monoclonal, Santa Cruz, USA; CK-MT antibodies: rabbit polyclonal, Abcam, USA); the blots were also labeled with glyceraldehyde-3-phosphate dehydrogenase (GAPDH) antibodies (Sigma-Aldrich, USA) to control for unequal loading. Relative levels of the three different CK isoforms were determined via densitometry analyses.

Total CK activity was measured spectrophotometrically with a CK diagnostic kit (Sigma, USA) as directed by the manufacturer’s instructions [7, 8]. Briefly, the frozen heart tissue was homogenized in ice-cold phosphate buffered saline (pH 7.4) and centrifuged at 10,000 g for 15 minutes; then, the protein content of the supernatant was measured via the Lowry method, and 10 μL of the supernatant was added to 100 μL of reconstituted reagent and incubated at 37°C. CK activity was measured by monitoring absorbance at 340 nm; one unit of CK was defined as the amount of enzyme that transferred 1 μM of phosphate from PCr to ADP per minute at pH 6.0.

### Statistical analysis

Data are presented as mean ± standard error. Significance (*P*<0.05) was evaluated via the Student’s *t* test for comparisons between two values and via analysis of variance (ANOVA) or repeated ANOVA and the Tukey posthoc test for comparisons between multiple values or repeated measurements. All statistical analyses were performed with SPSS software (version 13.0, SPSS, Chicago, USA).

## Results

### Differentiation of hiPSCs into CMs, ECs, and SMCs

**Fig 1:**
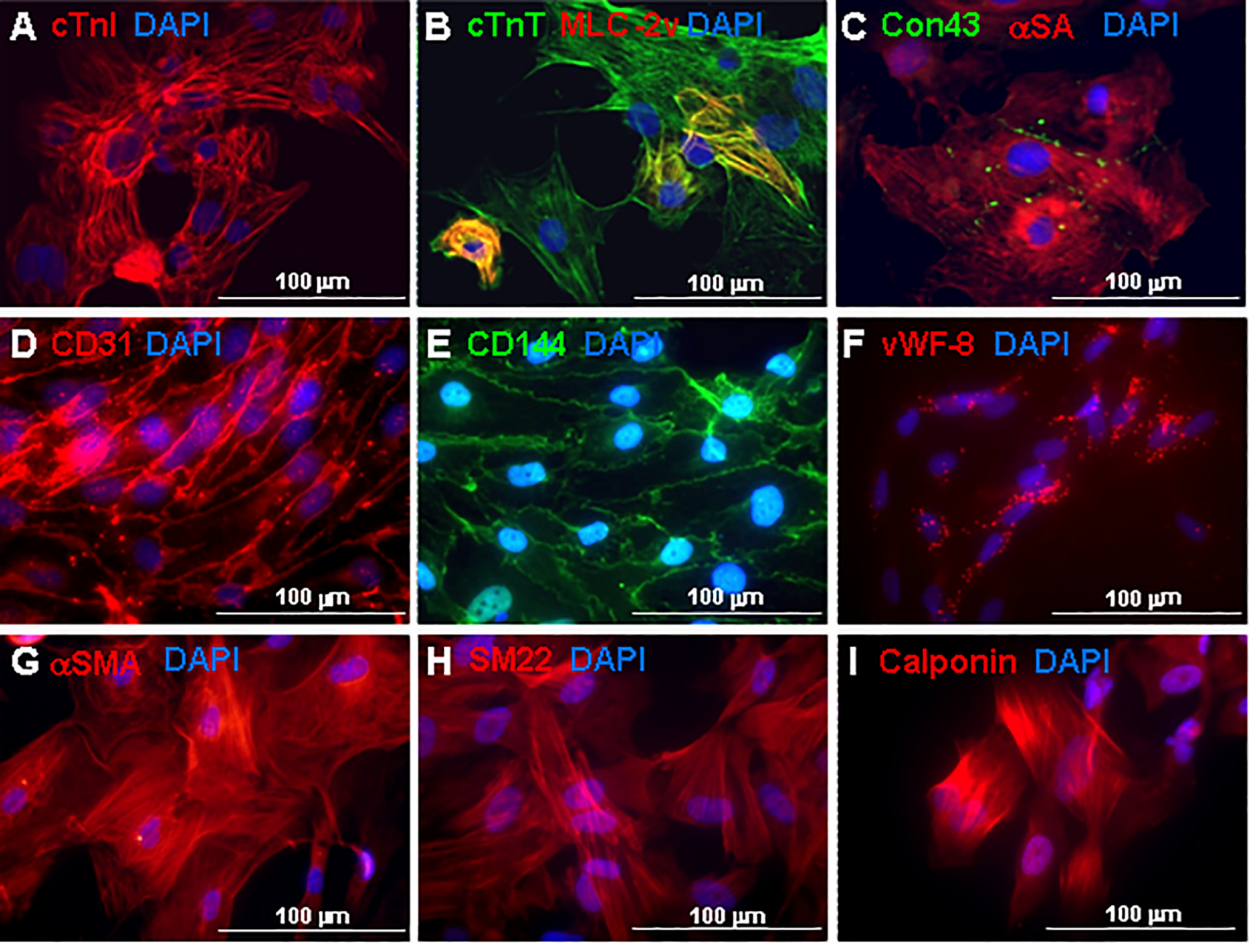
The differentiated hiPSC-CMs, -ECs, and -SMCs express appropriate lineage-specific markers. hiPSCs were differentiated into CMs, ECs, or SMCs via published protocols. (A-C) Expression of the CM-associated proteins (A) cardiac troponin I (cTnI, red), (B) cardiac troponin T (cTnT, green) and myosin light-chain 2v (MLC-2v, red), or (C) connexin 43 (Con43, green) and α sarcomeric actin (αSA, red) was evaluated in hiPSC-CMs; (D-F) expression of the EC-associated proteins (D) CD31 (red), (E) CD144 (green), or (F) von Willebrand factor 8 (vFW-8, red) was evaluated in hiPSC-ECs; and (G-I) expression of the SMC-associated proteins (G) α smooth-muscle actin (αSMA, red), (H) SM22 (red), or (I) calponin (red) was evaluated in hiPSC-SMCs. Assessments were performed via immunflourescence, and nuclei were counterstained with DAPI.

hiPSCs were reprogrammed from human neonatal dermal fibroblasts, engineered to express GFP, and then differentiated into CMs by modulating the Wnt/beta-catenin signaling pathway [14]. Contractions (S1 Video) typically began to appear 8 days after differentiation was initiated, and the number of contracting cells peaked on Day 12. The differentiated cells were purified and enriched for hiPSC-CMs by culturing them for 8 days in glucose-depleted culture medium with abundant lactate, and the expression of cardiac-specific proteins in the hiPSC-CMs was evaluated approximately one week after purification was complete. Nearly all of the hiPSC-CMs expressed cardiac troponin I (cTnI) (Fig 1A), cardiac troponin T (cTnT) (Fig 1B), and α sarcomeric actin (αSA) (Fig 1C), and cardiac connexin 43 was present at numerous points of contact between adjacent cells; 20%-30% of the hiPSC-CMs expressed the 2v isoform of myosin light-chain (MLC-2v) (Fig 1B), which is present only in ventricular CMs. hiPSC-ECs and hiPSC-SMCs were generated via established differentiation protocols [13, 15] and expressed EC-specific (CD31, CD144, and von Willebrand factor 8 [vWF-8]) (Fig 1D–1F) or SMC-specific (α smooth-muscle actin [αSMA], SM22, and calponin) (Fig 1G–1I) proteins, respectively.

### Combined treatment with a fibrin patch and injected hiPSC-derived cardiac cells partially improved the contractile indexes in infarcted swine hearts

**Fig 2:**
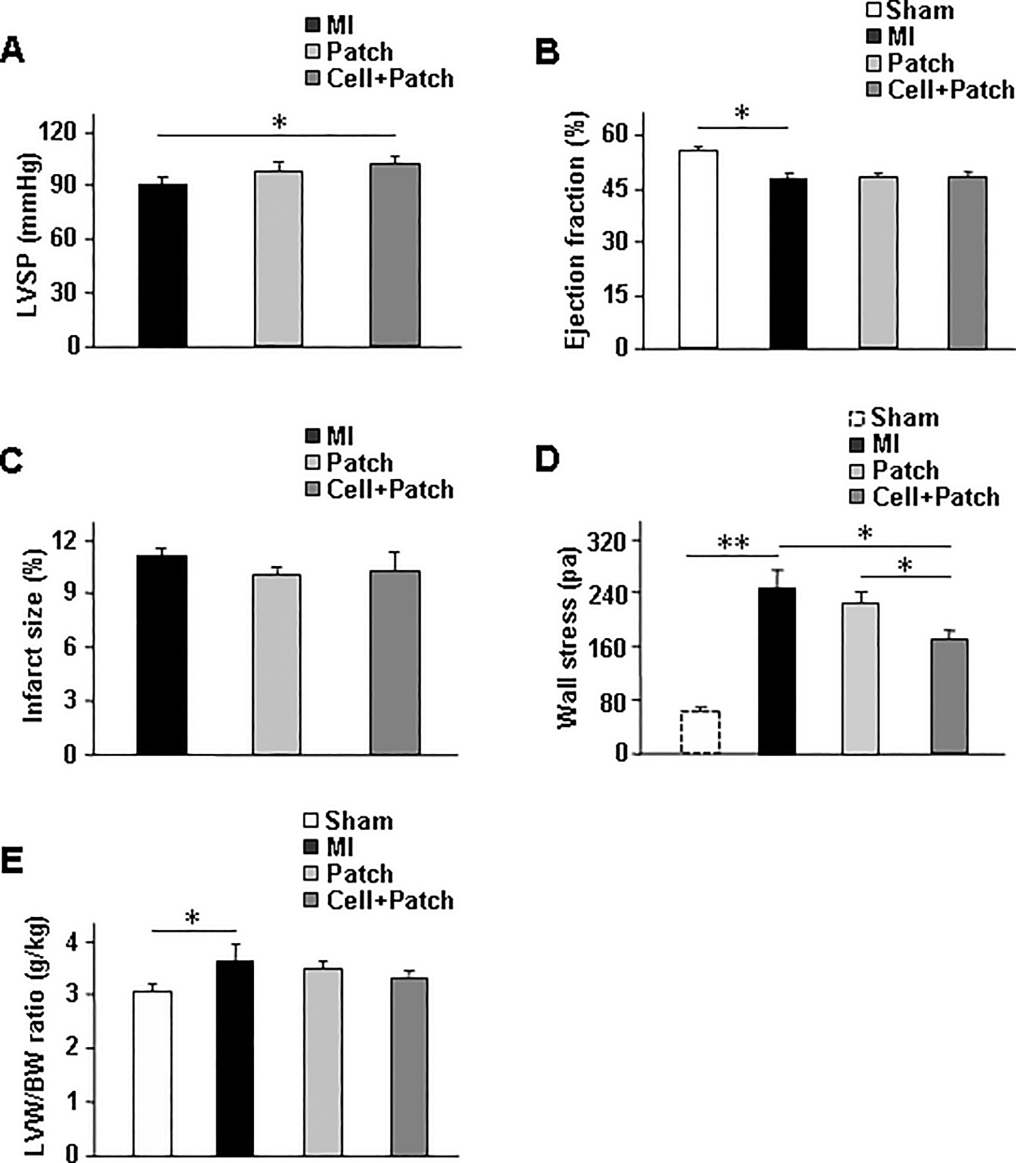
Measures of myocardial contractile activity changed in response to MI and to subsequent combined treatment with a fibrin patch and injected hiPSC-derived cardiac cells. Swine were treated with a fibrin patch and injected hiPSC-CMs, -ECs, and -SMCs (Cell+Patch), with the patch alone (Patch) or with neither experimental treatment (MI) after surgically induced myocardial infarction; the treatments were also withheld from a fourth group of animals than underwent sham surgery (Sham). (A) Hemodynamic measurements of the left-ventricular (LV) systolic pressure (SP), (B-C) MRI assessments of (B) the LV ejection fraction (EF) and (C) infarct size, (D) end-systolic LV wall stress of the infarct region, and (E) the LV-weight to bodyweight (LVW/BW) ratio were evaluated four weeks after injury. *P<0.05; n = 5–6 per experimental group.

Myocardial infarction (MI) was induced via surgical IR injury in swine. A fibrin patch was created over the site of infarction (S2 Video) in animals from the Patch and Cell+Patch groups, and 6 million hiPSC-derived cardiac cells (2 million hiPSC-CMs, 2 million hiPSC-ECs, and 2 million hiPSC-SMCs) were injected into the injured myocardium of animals in the Cell+Patch group via a needle inserted through the patch. MI was also induced in a third group of animals (i.e., the MI group) that received neither the patch nor the injected cells after injury, while animals in the Sham group underwent all surgical procedures for MI induction except the ligation step and recovered without either experimental treatment. Four weeks after injury, hemodynamic measurements of left ventricular systolic pressure (LVSP) were significantly greater in Cell+Patch animals comparing to animals in MI group (Fig 2A). Cardiac function and infarction size were evaluated by cardiac MRI. Comparing to the sham animals, MI animals showed significantly reduced left-ventricular ejection fraction (LVEF) and increased LVW/BW ratio (Fig 2B and 2E). Infarct regional wall stress was significantly lower in Cell+Patch heart than in MI and patch hearts (Fig 2D).

### Combined treatment with a fibrin patch and injected hiPSC-derived cardiac cells improved cellular energy metabolism in infarcted swine hearts

**Fig 3:**
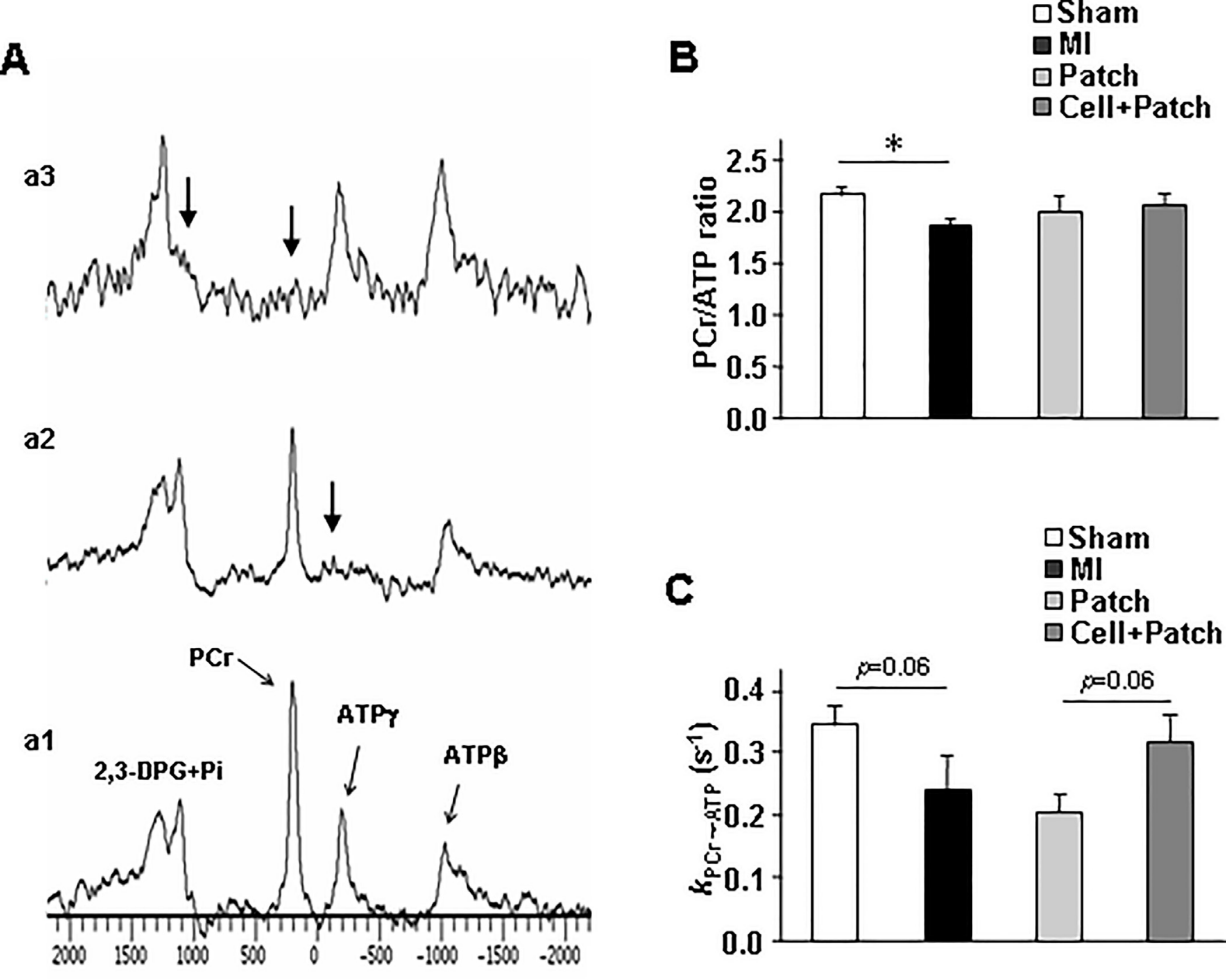
Global assessments of the myocardial bioenergetics of infarcted swine hearts changed in response to combined treatment with a fibrin patch and injected hiPSC-derived cardiac cells. (A) Global assessments of myocardial ATP metabolism were evaluated via an established ^31^P MRS-MST experiment. The experiment consists of a control spectrum obtained without saturation (**a1**) to quantify the magnetization (M0) of phosphocreatine (PCr) and ATPγ, a spectrum obtained with ATPγ saturated (**a2**) to quantify the steady-state magnetization (Mss) of PCr, and a spectrum obtained with both Pi and PCr saturated (**a3**) to quantify the steady-state magnetization of ATPγ; saturation was performed with BISTRO frequency-selective pulses applied at the positions indicated by the arrows in spectra **a2** and **a3**. This approach enables the rates of ATP utilization to be calculated in vivo, even when the resonance for inorganic phosphate (Pi) is obscured by the resonance of 2,3-diphosphoglycerate (2,3-DPG). (B-C) Spectra a1, a2, and a3 were used to calculate global measures of (B) the PCr/ATPγ ratio, and (C) the rate of the PCr→ATP reaction (*k*_PCR→ATP_) in the hearts of animals in the Cell+Patch, Patch, MI, and Sham groups. *P<0.05; n = 4–5 per experimental group.

**Fig 4:**
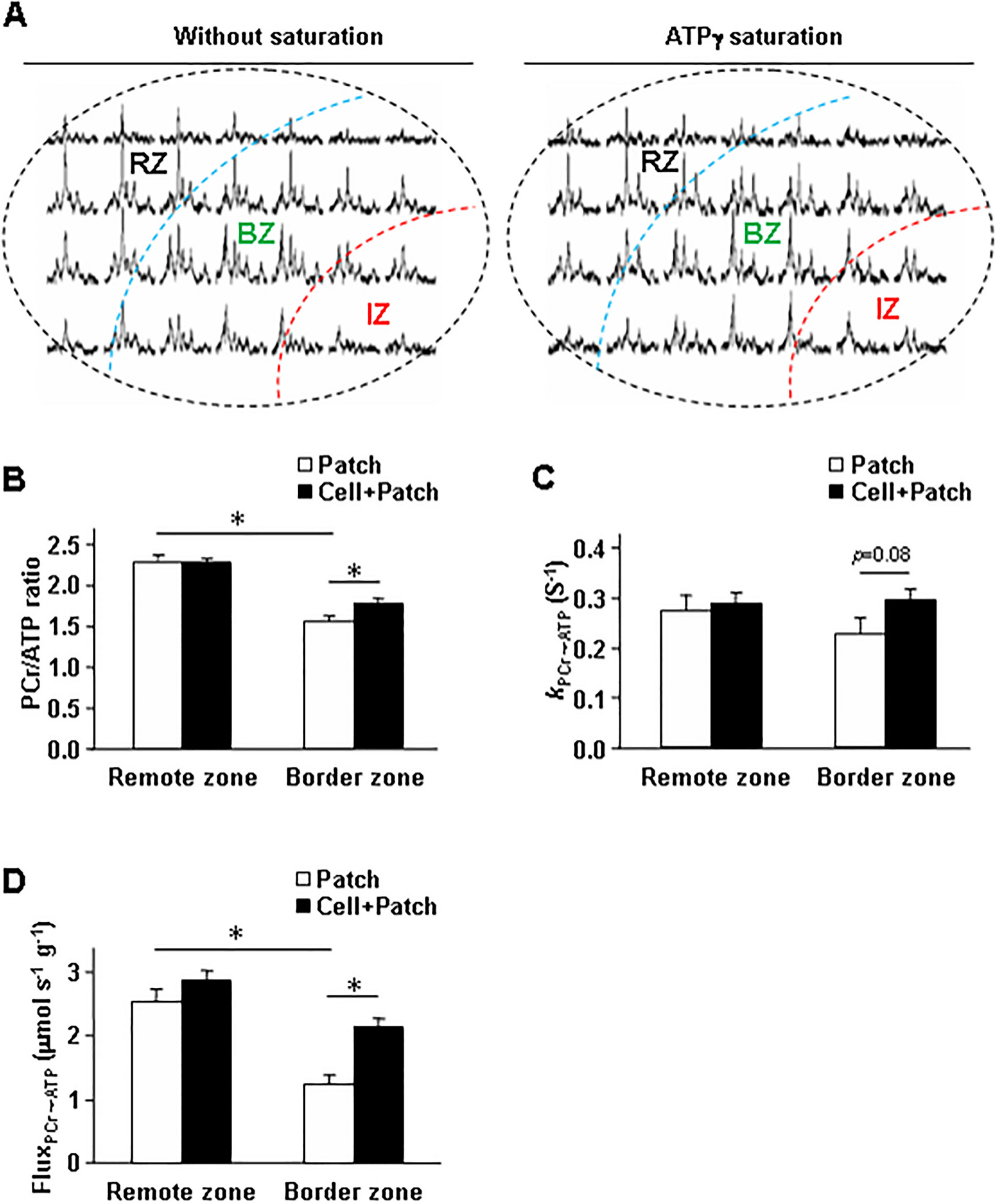
Spatially localized assessments indicate that the myocardial bioenergetics of swine hearts are disrupted at the border zone of infarction and partially restored by combined treatment with a fibrin patch and injected hiPSC-derived cardiac cells. (A) Spatially localized assessments of myocardial energetics were performed via ^31^P 2D-CSI MRS. Spectra were obtained both with (right) and without (left) saturation of the ATPγ resonance and used to calculate (B) the PCr/ATPγ ratios and (C) the rate of the PCr→ATP reaction (*k*_PCR→ATP_) at the border-zone of infarction and in remote (i.e., noninfarcted) myocardial regions from the hearts of animals in the Cell+Patch and Patch groups. (D) The Flux for the PCr→ATP reaction (Flux_PCr→ATP_), as the product of multiplying *k*_PCR→ATP_, PCr/ATP ratios, and the ATP level that measured by chemical method. *, *P*<0.05; n = 4–5 per experimental group.

The CK reaction is the prime energy reserve of the heart, as it rapidly and reversibly converts PCr and ADP to ATP and creatine. We performed ^31^P MRS-MST experiments (Fig 3A) to determine whether indices of myocardial energy metabolism differed among animals in the Sham, MI, Patch, and Cell+Patch groups at Week 4 after injury. While the PCr/ATP ratio was similar in Sham, Patch, and Cell+Patch animals, it was significantly lower in MI animals than in Sham animals (Fig 3B). The value of *k*_PCr→ATP_ was lower in MI animals than in Sham animals, and was significantly greater (*p* = 0.06) in Cell+Patch animals than in Patch animals (Fig 3C). Moreover, when assessments were spatially localized via 2D-CSI MRS (Fig 4A), border-zone measurements of both PCr/ATP (Fig 4B) and Flux_PCr→ATP_ (Fig 4D) were significantly greater in Cell+Patch animals than in Patch animals, and differences between the two groups for border-zone measurements of *k*_PCr→ATP_ (Fig 4C) approached significance (*p* = 0.08). The PCr/ATP ratio and Flux_PCr→ATP_ values were also significantly lower in the border-zone than in remote myocardium from only patch animals (Fig 4B and 4D).

**Fig 5:**
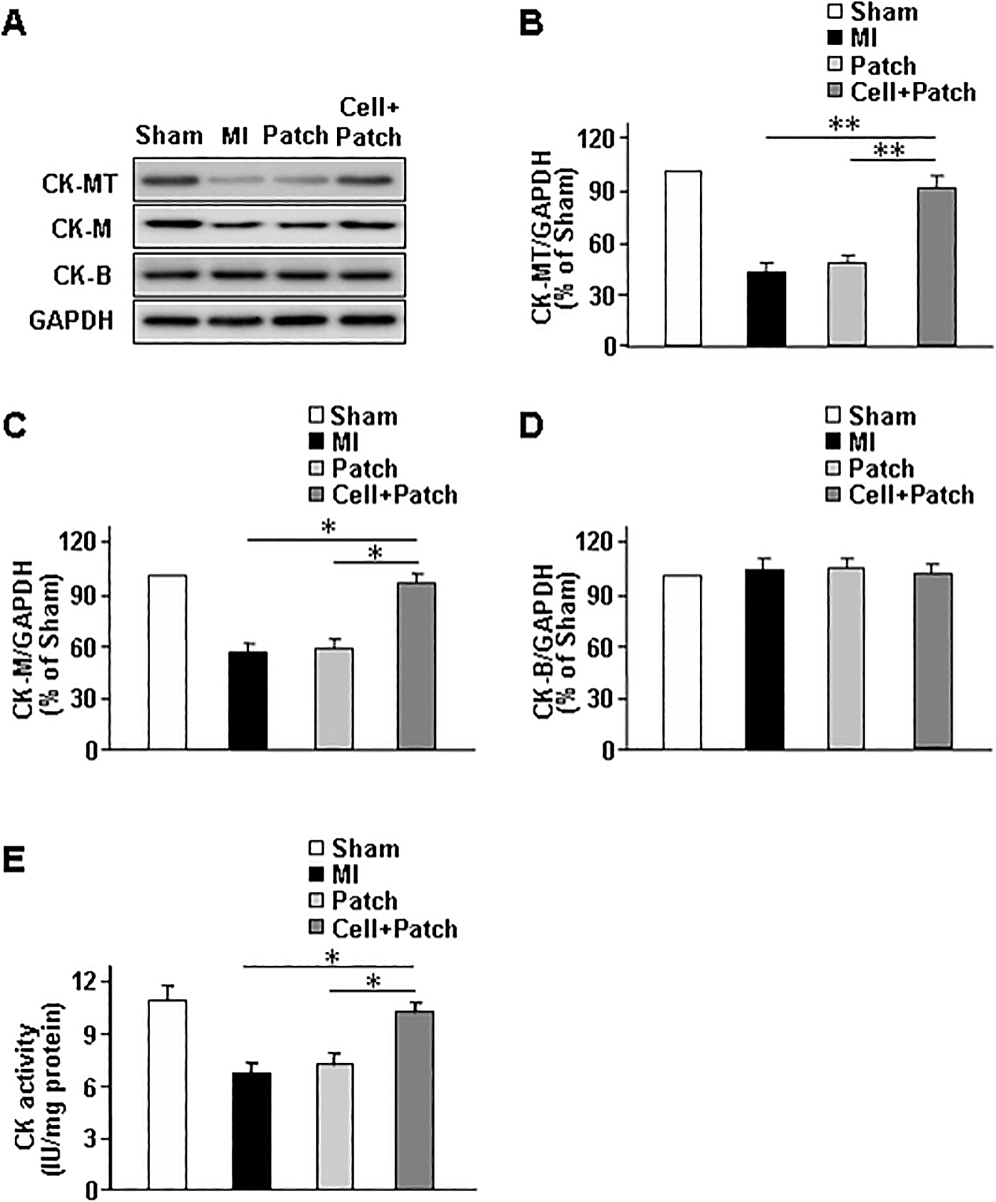
Injected hiPSC-derived cardiac cells increased CK-MT and CK-M levels and total CK activity. Myocardial tissue was harvested from the border zone of infarction in Sham, MI, Patch, and Cell+Patch animals. (A) The harvested tissues were analyzed via Western-blot to determine the amount of protein present for each of three creatine kinase isoforms (CK-MT: mitochondria, CK-M: myocardium, CK-B: brain) and for glyceraldehyde-3-phosphate dehydrogenase (GAPDH). (B) CK-MT, (C) CK-M, and (D) CK-B protein levels were quantified via densitometry, normalized to GAPDH protein levels, and presented as a percentage of the amount present in Sham animals. (E) Total CK activity in the harvested tissues was measured spectrophotometrically. *P<0.05, **P<0.01.

The results from our 2D-CSI MRS assessments were corroborated by quantifying CK levels in the BZ of infarction. Western blot analyses indicated that both CK-MT and CK-M were significantly less abundant in the BZ of hearts from animals in the MI and Patch groups than in the BZ of hearts from Cell+Patch animals (Fig 5A–5C), while BZ levels of CK-B in all three groups were similar (Fig 5D). Spectrophotometric measurements of global CK activity were also significantly lower in the BZ of MI and Patch animals than in the corresponding region of hearts from animals in the Cell+Patch group (Fig 5E). Collectively, these observations suggest that our 2D-CSI MRS technique can successfully detect myocardial bioenergetic abnormalities in specific regions of infarcted swine hearts, and that these abnormalities may be at least partially corrected by treatment with a fibrin patch and injected hiPSC-derived CMs, ECs, and SMCs.

## Discussion

A variety of cell types and administration techniques have improved cardiac function in both clinical trials and animal models of cardiovascular disease [28–32]; however, the mechanisms for any treatment-related effects have yet be characterized. Deficiencies in cellular ATP machinery and CK forward ATP flux systems have long been thought to contribute to the progressive declines in myocardial function that occur in patients with left ventricular hypertrophy (LVH) [4, 33]. The abnormality in CK system is most severe in hearts with coexistent of severe LVH and heart failure [5, 7]. However, the important concept of heterogeneity in regional CK flux abnormality in heart with post infarction LV remodeling has been impossible to investigate in vivo because of the very long data acquisition time to measure a CK forward rate constant (*k*_**PCr**→**ATP**_) [1, 10]. Xiong, et al., developed a set of ^31^P MRS pulse and pulse sequence methods for fast measuring bioenergetic parameters in the hearts of living animals [6, 9, 19, 25]. Particularly, the T_1_^Nom^ methods that effectively reduced the *k*_**PCr**→**ATP**_ data acquisition time by 82% [9]. For the experiments presented in this report, we combined T_1_^Nom 31^P MRS-MST with 2D-CSI, which enabled us to monitor myocardial CK forward rate constant in the BZ and RZ of the heart with postinfarction LV remodeling during a single acquisition period and without surgically moving the MRI surface coil. The novel findings are that the BZ CK flux rate is significantly lower than that in RZ, and this deficiency can be partially alleviated by treatment with a fibrin patch combined with injected hiPSC-derived cardiac cells. Furthermore, this bioenergetic improvement is accompanied by the protein expression level and total activity of CK isozyme. The CK-m and CK-mt levels, as well as the total CK activity, were significantly greater in BZ tissues in Cell+Patch treated hearts than those in BZ myocardium of MI hearts.

Although the Cell+Patch therapy investigated here was not associated with significant improvements in measurements of cardiac function or infarct size, similar experiments in a larger group size of number of animals (n = 11–12 per group versus n = 5–6 in the current study) suggested that combining patch application with injected hiPSC-ECs, -SMCs, and -CMs led to significant improvements in both parameters [12]. Furthermore, the patch used in the previous investigation contained microspheres that secreted insulin-like growth factor over an extended period, which may have protected the transplanted cells from apoptosis. Thus, the lack of significance observed in our current study can likely be attributed to the smaller number of animals included in the investigation and, perhaps, to a somewhat lower rate of survival for the transplanted cells.

In conclusion, the experiments presented in this report demonstrate that our 2D-CSI protocol can successfully evaluate myocardial ATP flux rate via CK in multiple regions of the same heart from data collected during a single acquisition period and from a single coil probe location. We also show that combined treatment with a fibrin patch and injected hiPSC-derived cardiac cells may partially correct the bioenergetic abnormalities associated with IR injury by increasing the rate and flux of CK-mediated ATP production. Notably, some evidence suggests that bioenergetic parameters, such as the PCr/ATP ratio or *k*_PCr→ATP_, can be used to predict the mortality of patients with HF [4–7], so the NMR method established in the present study may prove to be a useful tool for monitoring the progress of LV dysfunction, or in response to therapeutic interventions.

## Supporting Information

S1 Video A sheet of hiPSC-CMs was shown 5 days after contractions were first observed. (AVI)

S2 Video A fibrin patch was created over the site of pig ischemic myocardium. (AVI)
